# Delayed Management of Zollinger-Ellison Syndrome in a Noncompliant Patient

**DOI:** 10.7759/cureus.8471

**Published:** 2020-06-06

**Authors:** Artsiom Klimko, Oana Plotogea, Alexandru Constantinescu, Gabriel Constantinescu

**Affiliations:** 1 Division of Physiology and Neuroscience, Carol Davila University of Medicine and Pharmacy, Bucharest, ROU; 2 Department of Gastroenterology, Carol Davila University of Medicine and Pharmacy, Clinical Emergency Hospital of Bucharest, Bucharest, ROU; 3 Department of Gastroenterology and Hepatology, Carol Davila University of Medicine and Pharmacy, Floreasca Emergency Hospital, Bucharest, ROU

**Keywords:** zollinger-ellison syndrome, gastrinoma, necrotic duodenitis, duodenal ulceration, noncompliance

## Abstract

We present a case of a 60-year-old male diagnosed with Zollinger-Ellison syndrome (ZES) after a protracted multicentric workup for chronic diarrhea and unexplained weight loss. ZES is intrinsically difficult to diagnose due to nonspecific symptoms, which are mimicked by other more frequent pathologies, such as peptic ulcer disease secondary to Helicobacter pylori or nonsteroidal anti-inflammatory drugs. The diagnostic challenge can be further complicated by patient noncompliance, resulting in delayed management and unnecessary health care. In our case report, the patient did not adhere to the care plan preceding endoscopy and failed to maintain communication with the treating doctor. As a result, crucial information was missing, and establishing the diagnosis of ZES took six months. Delay in appropriate management also contributed to poor disease course, heavy necrotic ulceration of the duodenum and proximal jejunum that was discovered on repeat endoscopy.

## Introduction

Zollinger-Ellison syndrome (ZES) is caused by gastrin secreting neuroendocrine tumor (NET), aptly named gastrinoma, which results in severe peptic ulcer disease and chronic diarrhea. Research about ZES peeked in the 1970s, when advances in physiology and imaging furthered our knowledge about the syndrome. Since 1955, thousands of articles have been published and despite being a rare pathology, it is widely accepted that physicians are well acquainted with ZES. However, diagnosis is becoming increasingly difficult to make due to the nonspecific symptoms which are further confounded by high incidence of other pathologies that have similar symptomatology, namely peptic ulcer disease caused by Helicobacter pylori or nonsteroidal anti-inflammatory drug (NSAID) use [1,2].

Symptomatic treatment with proton pump inhibitors (PPI) has contributed to complicating the diagnosis by masking symptoms, as studies have found decreasing rates of diagnosis of ZES when diagnosis rates have been compared to pre- and post-PPI periods [3,4]. Predictably, these decreasing diagnosis rates delay detection and explain the resurgence of advanced metastatic disease [5]. Roy et al. found that upon initial referral, as little as 3% of physicians were able to correctly diagnose ZES, leading to a delay in diagnosis that approaches five years [6]. This means that, currently, the most common cause of morbidity and mortality in patients with ZES is metastatic disease [7]. In this context, we present a patient with ZES in whom the diagnosis was delayed by six months due to a common, but overlooked problem, patient noncompliance. Additionally, we present and explore the endoscopic findings of ZES to potentially prepare gastroenterologists, as many are unlikely to encounter this pathology frequently throughout their medical career.

## Case presentation

A 60-year-old male was referred to our gastroenterology department at Floreasca Hospital after a six-month interdisciplinary and multicentric workup for a two-year history of diarrhea and unexplained weight loss. At this time, he was referred by an infectious diseases department after complex explorations for diarrhea of presumably infectious etiology. Upon admission to our department, chief complaints included unrelenting secretory diarrhea with up to 20 watery bowel movements per day, a total weight loss of 35 kilograms in nine months, and intermittent epigastric pain. The physical exam was unrevealing. Pertinent laboratory findings upon admission included leukocytosis (20,300/μL) with neutrophilic predominance and a mild normocytic normochromic anemia (hemoglobin 11.9 g/dL). It is important to establish that at this point, the patient was already followed up extensively at three hospitals and the treatment regimen included rifaximin, otilonium bromide, trimebutine, loperamide, antacids, PPIs, antiemetics, NSAIDs, dexamethasone, pancreatic enzyme replacement, trimethoprim/sulfamethoxazole, and a probiotic. Past medical history included grade I hypertension, type 2 diabetes mellitus, psoriasis, and gout.

The initial workup began six months prior. Despite a long-standing history of secretory diarrhea, the patient only sought medical attention when he became alarmed by unexplained weight loss (from 100 to 80 kilograms in several months). Bowel movements were watery, without blood or mucus, they were not associated with vomiting, fever, or heartburn, and there was no recent travel history.

Over the next two months, the patient was followed up at endocrinology and gastroenterology departments to establish a diagnosis. The initial workup is summarized in Table 1. Two investigations were not completed successfully, namely the esophagogastroduodenoscopy (EGD) and the chromogranin A (CgA) assay. The patient did not adhere to the care plan and ate the morning of the scheduled EGD. As a result, only the stomach and first part of the duodenum were visualized, missing the duodenal ulceration that was likely present. The colonoscopy revealed external hemorrhoids and diverticulitis, without any other abnormalities.

**Table 1:** Laboratory tests performed during the initial workup at the gastroenterology and endocrinology departments. N: normal; C. difficile: Clostridioides difficile; HIV: human immunodeficiency virus; TSH: thyroid-stimulating hormone; fT3: free T3; fT4: free T4; CEA: carcinoma embryonic antigen; CA19-9: carbohydrate antigen 19-9; CA125: carbohydrate antigen 125; 5-HIAA: 5-hydroxyindoleacetic acid.

Investigation completed	Result of the investigation
Erythrocyte sedimentation rate	16 mm/hr (N = 1–13 mm/hr)
C. difficile toxin assay	Negative
HIV antigen/antibody test	Negative
Thyroid hormone profile (TSH, fT3, fT4)	Within normal limits
Tumoral markers (CEA, AFP, CA19-9, CA125)	Within normal limits
5-HIAA	7.8 mg/dL (N = 2–9 mg/dL)
Serum serotonin	249 μg/dL (N = 80–400 μg/dL)
Serum chromogranin A	1,880 μg/L (N = 27–94 μg/L)

Despite the problematic EGD, the suspicion for an NET was still present and CgA levels, in addition to serum serotonin and urinary 5-hydroxyindoleacetic acid, were ordered. The patient completed the ordered tests, which later revealed an elevated CgA (1,880 μg/L. Unfortunately, he did not notify or maintain correspondence with the treating doctor because he became frustrated with the seemingly futile barrage of investigations. The crucial piece of information was discovered only months later at our gastroenterology department after CgA levels were ordered again. The patient recalled he has completed this test before and presented the relevant paperwork. With an inconclusive EGD and a yet unrevealed CgA elevation, a diagnosis of ZES was difficult to support at this time. Despite dietary modifications and symptomatic treatment in the following weeks, the patient continued to lose weight and diarrhea was now accompanied by episodes of vomiting.

He decided to seek treatment with an infectious diseases department at a regional tertiary care center.

Upon admission, the pertinent laboratory values included leukocytosis with neutrophilic predominance, thrombocytosis, and elevated fibrinogen. During the next month, another extensive workup was performed (Table 2) and predictably did not yield any definitive information. Worsening heartburn and newly found melena in the patients’ stool prompted a computed tomography (CT) scan of thorax and abdomen, which was largely inconclusive, only finding some inflammatory changes in the first loops of the jejunum. In light of these findings, the patient was transferred to a gastroenterology department at our hospital.

**Table 2:** Laboratory tests performed at the infectious disease department. N: normal; IgA anti-tTG: immunoglobulin A anti-tissue transglutaminase; IgG DGP: immunoglobulin G deamidated gliadin peptide; C. difficile: Clostridioides difficile; ELISA: enzyme-linked immunosorbent assay; HIV: human immunodeficiency virus; HTLV: human T-cell lymphotropic virus; CMV: cytomegalovirus.

Investigation completed	Result of the investigation
Erythrocyte sedimentation rate	24 mm/hr (N = 1–13 mm/hr)
Calprotectin	9.88 mg/L (N ≤ 10 mg/L)
Celiac disease tests (IgA anti-tTG and IgG DGP)	Negative
Lactose intolerance (hydrogen breath test)	Within normal limits
C. difficile toxin assay (repeated)	Negative
ELISA for Toxoplasma gondii and Toxocara canis	Negative
Tests for HIV, HTLV, CMV, adenovirus, rubella, hepatitis (A, B, C)	Negative
Borrelia burgdorferi	Ongoing at the time of admission

**Figure 1:**
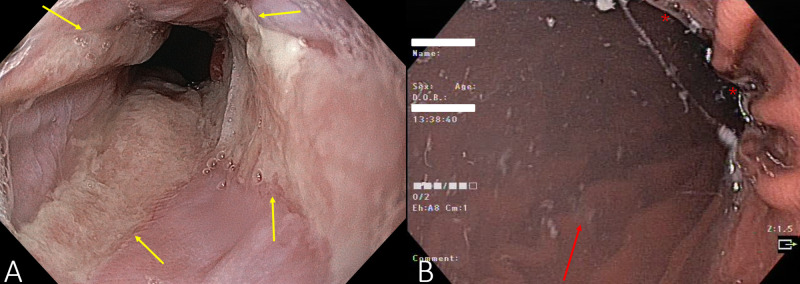
Endoscopy of the distal esophagus (A) showing severe reflux with circumferential ulcerations present in four quadrants (yellow arrows) and the stomach (B) showing increased secretions (marked with red stars) and hypertrophic gastric folds (red arrow showing one well delineated fold in the background).

The workup at our gastroenterology department began with repeat high-quality EGD and colonoscopy. This time the EGD revealed multiple abnormalities. The lower two-thirds of the esophagus were covered with linear erosions in all four quadrants (Figure 1A), leading to the diagnosis of esophagitis graded Los Angeles D [8]. The stomach had an excess of secretions and hypertrophic folds (Figure 1B).

**Figure 2:**
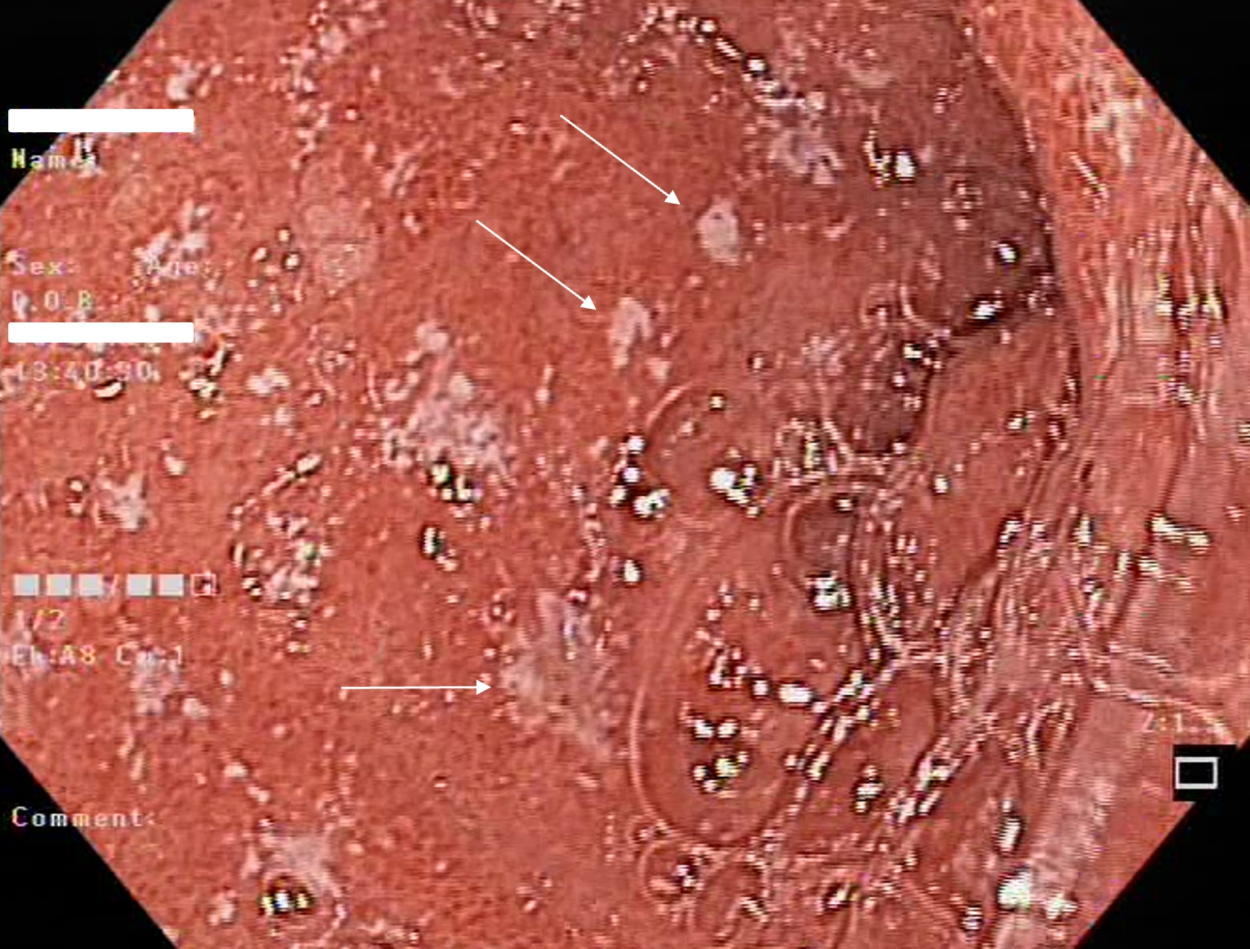
Endoscopy of the duodenal bulb showing dozens of ulcerations (arrows) approximately several millimeters in size.

The duodenal bulb had dozens of ulcerations several millimeters in size (Figure 2). 

**Figure 3:**
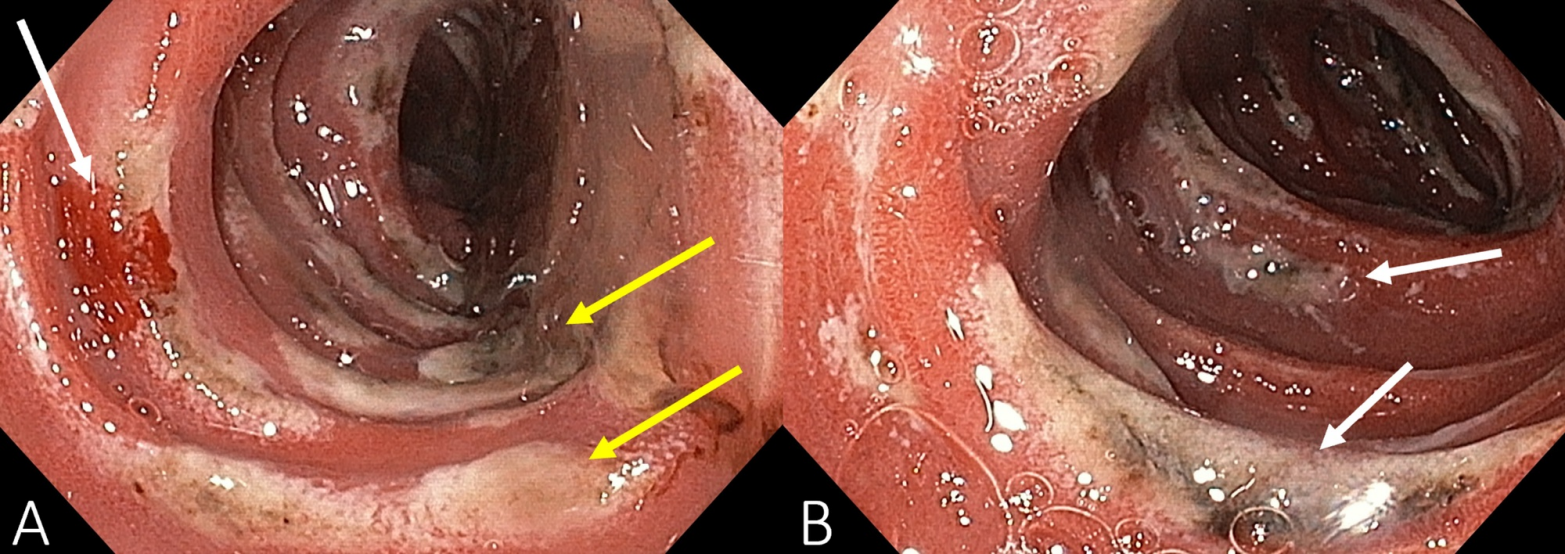
Endoscopy of the second portion of the duodenum (A) showing necrotic ulcerations (yellow arrows), which began to bleed when lightly touched with the endoscope (white arrow) and the duodeunojejunal flexure (B) with more necrotic ulcers (white arrows).

The second portion of the duodenum was covered in necrotic ulcerations, which began to bleed when lightly touched with the endoscope (Figure 3A). At the duodenojejunal flexure, more necrotic ulcers were found (Figure 3B). The patient’s serum gastrin was measured, and he was started on IV omeprazole (40 mg) every six hours, which completely resolved the symptoms and decreased the leukocyte count from 23,000/μL to 12,000/μL. Serum gastrin was found to be 450 pg/mL (N = 13-115 pg/mL). Calcitonin, phosphate, calcium, prolactin, and insulin-like growth factor-1 were all normal, ruling out multiple endocrine neoplasia type 1 (MEN-1), and confirming sporadic ZES. 

**Figure 4:**
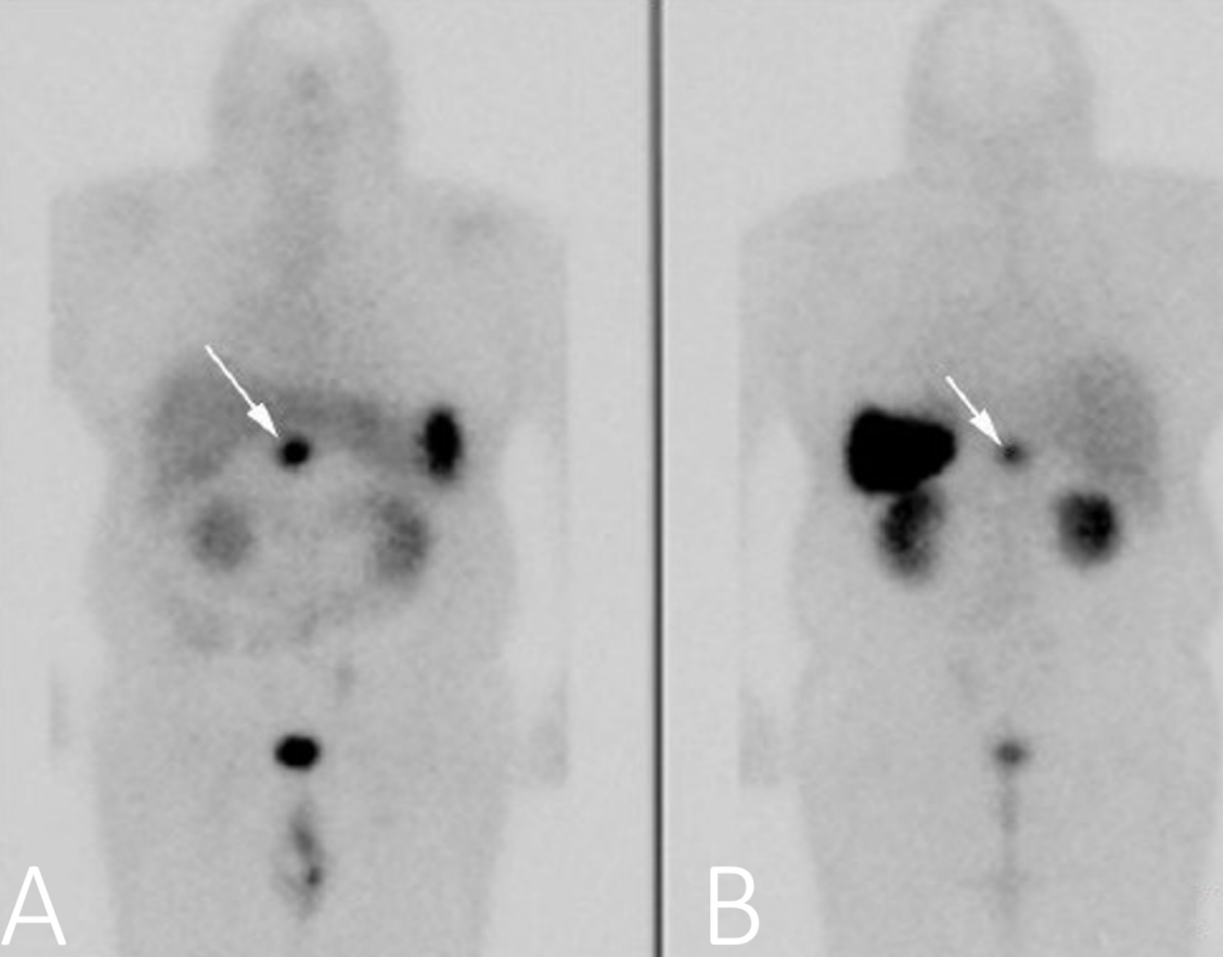
Somatostatin receptor scintigraphy: anteroposterior (A) and posteroanterior (B) view four hours after contrast administration demonstrating intense focal uptake in the area of the third portion of the duodeunum. Also note the physiologic uptake of contrast into the spleen, kidneys, and bladder.

Abdominal MRI with contrast was done; aside from revealing diffuse thickening of the gastric wall and peripancreatic lymphadenopathy measuring approximately 2 cm, the tumor (or metastasis) was not visible. As was discovered later, the peripancreatic lymphadenopathic mass (which we considered to be an inflammation secondary to necrotic duodenitis) was in fact the NET we were looking for, but at that time we did not give it significance. Even the endoscopic ultrasound performed during hospitalization did not raise any suspicion for that pathology. Somatostatin receptor scintigraphy (SRS) with indium-11 was performed, and besides physiologic uptake of contrast in the kidneys and spleen, the scan revealed intense focal uptake in a 28 x 17 mm nodule localized in the superior surface of the third portion of the duodenum (Figure 4).

**Figure 5:**
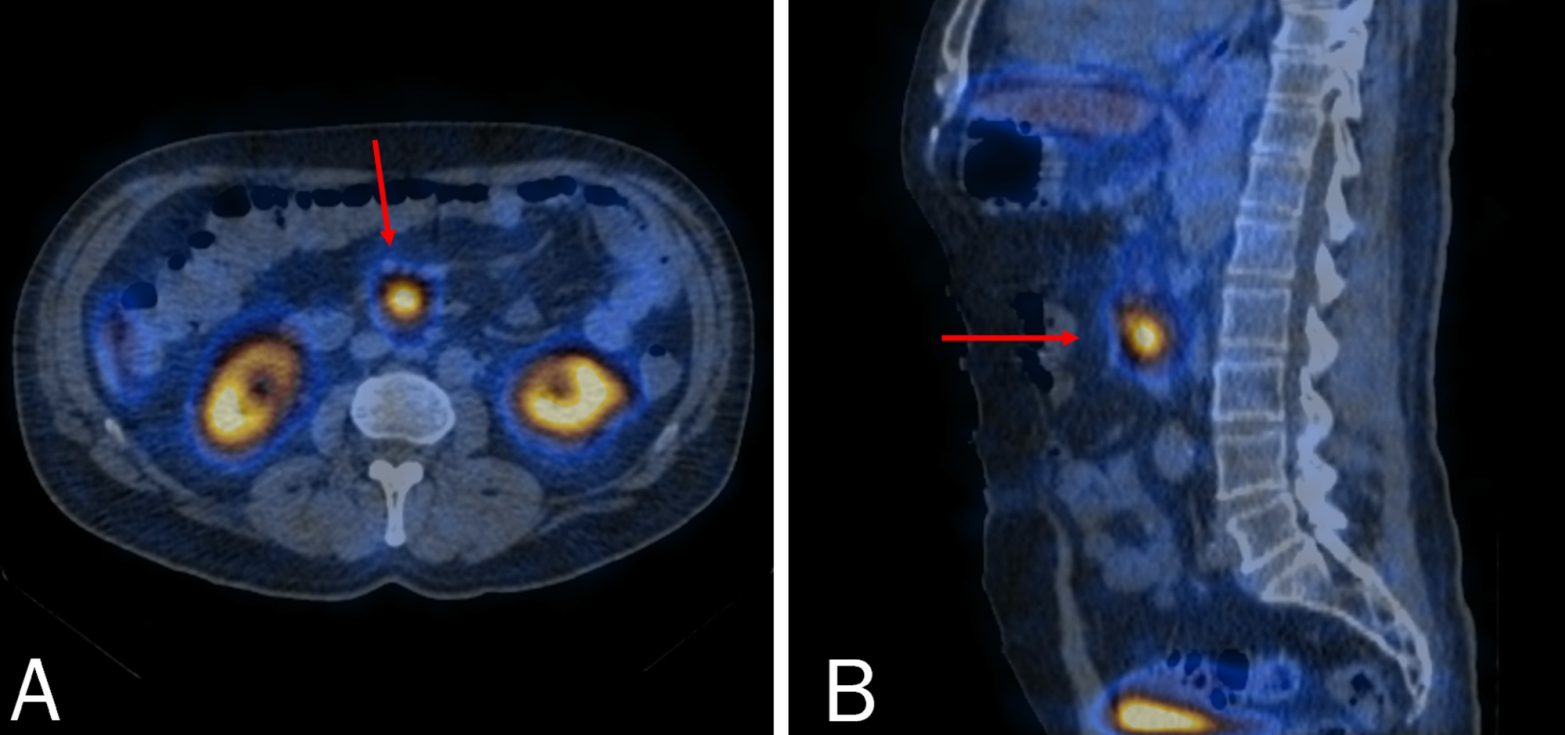
SPECT: transverse (A) and sagittal (B) views demonstrating focal uptake of contrast (arrows) behind the head of the pancreas, adjacent to the third portion of the duodenum. SPECT: single-photon emission computerized tomography.

To obtain precise localization of the NET, whole-body single-photon emission computerized tomography (SPECT) with 99mTc-Tektrotyd was ordered to confirm the diagnosis of solitary somatostatin-receptor positive gastrinoma located behind the pancreatic head adjacent to the third portion of the duodenum (Figure 5). The patient was successfully maintained on high-dose esomeprazole for three months until undergoing curative Whipple pancreaticoduodenectomy. Prior to the resection, the serum CgA was noted to be 817 μg/L. The postoperative period was uneventful and the patient was discharged on post-operative day 16. During follow-up one month later, the patient was found to be doing well, with complete resolution of symptoms and good intestinal transit. 

## Discussion

ZES is frequently discussed, but rarely encountered. The annual incidence of ZES is estimated to be one to three per million, caused by an NET secreting gastrin, with subsequent severe hyperchlorhydria driving the symptomatology [9]. A number of the symptoms present in our patient were suggestive of ZES, namely chronic diarrhea, abdominal pain, gastroesophageal reflux disease (GERD), and severe peptic ulcer disease; together, these symptoms are seen in up 45%-70% of patients with ZES [6,10]. Without much useful information obtained from the first EGD, the elevated CgA level would have facilitated an earlier diagnosis. More attention should have been given to the physician-doctor relationship to establish the source of noncompliance. Whether it was a lack of education about the scheduled procedures or the exasperation secondary to the protracted hospital course, identifying and addressing these barriers would have made a significant difference. Ultimately, the three crucial steps that allowed use to make the diagnosis included the following: abnormalities found on upper digestive endoscopy, abnormal gastrin level, and diagnostic octreoscan.

Discovering the elevated CgA sooner would have allowed to steer the initial workup towards ZES. CgA is a glycoprotein secreted from dense core granules of neuroendocrine cells, which can be used as a tumor marker for NETs [11]. Many iatrogenic, oncologic, and nononcologic conditions have been proven to raise CgA levels, and this, coupled with the fact that as many as 50% of NETs do not even secrete this protein, hinders the sensitivity of CgA [12]. Gastrinomas have been associated with a much higher CgA value, within the context of typical cutoffs ranging from 84 to 87 μg/L, and mean CgA values in ZES have been reported to be as high as 1,490 μg/L [13]. Since gastrinomas are associated with much higher levels of CgA, the clinical utility of this marker may be underappreciated.

With a CgA cutoff of 78.7 μg/L, Hijoka et al. found that as many as 94% of gastrinoma patients had elevated CgA; in other words, the usefulness of CgA levels in gastrinomas may be somewhat obscured because it is reported for all NETs as a whole, rather than distinguishing gastrinomas from other NETs [14]. This is an important point to discuss because when CgA levels are aggregated together from patients with different types of NETs (e.g., gastrinomas, glucagonomas, insulinomas), the sensitivity (53.6%-71.1%) and specificity (78.6%-93%) of CgA are relatively low [13-15]. However, CgA should still not replace assays for the specific peptide hormones produced by an NET.

Ultimately, evidence is somewhat conflicting, and if there is suspicion specifically for a gastrinoma, workup should begin with measuring serum gastrin and gastric pH rather than CgA [16]. For our patient, it was interesting to see the gastrin level (450 pg/mL) to be relatively low (<10x upper limit of normal) despite the presence of relatively large tumor. Due to the unequivocal EGD findings, a secretin stimulation test was not performed, but in other circumstances it may be necessary, as serum gastrin can rise as high as 241 and 341 pg/mL with standard-dose and high-dose PPI, respectively [11].

The repeat EGD findings are somewhat unique, usually the ulcerations seen in ZES are less than <1 cm in diameter and over 75% are localized to the proximal duodenum, with less than 14% extending to the distal duodenum and 11% to the jejunum [9]. In another study of 72 patients, Wilcox et al. reported that as little as 5.6% and 2.8% gastrinomas presented with duodenal and jejunal erosions, respectively [10]. The protracted course of the disease and delayed management likely contributed to the heavy duodenal ulceration seen in our patient. Despite high-grade GERD and heavy ulceration being infamous hallmarks of ZES, we would like to draw attention to the hypertrophic gastric folds, which have been consistently proven to be the most common (>85% of patients) endoscopic findings in this disease [6,10]. The tumor was located within the gastrinoma triangle on the posterior surface of the head of the pancreas adjacent to the third portion of the duodenum, hence necessitating SPECT to localize [17].

The clinical utility, sensitivity, and specificity of imaging for anatomic localization of gastrinomas are predominantly dependent on tumor size. Most recent guidelines recommend the use of endoscopic ultrasound to begin the localization process for pancreatic tumors >2 cm, as its sensitivity (75%-100%) and specificity (95%) are higher than that of CT or MRI [18]. As illustrated in our case, even in context of relatively large tumors (28 x 17 mm), detection with CT/MRI and even endoscopic ultrasound may be difficult. The SPECT-SRS imaging protocol used to detect the tumor in our patient was determined by Sainz-Esteban et al. to have high sensitivity (88%) and specificity (97%) in a study of 103 patients with NETs [19]. In a study of 88 patients, Stokkel et al. reported that CgA and SRS may be combined to achieve a 90% sensitivity when used for follow-up of NETs and would be an acceptable modality to monitor for recurrence in our patient [15].

## Conclusions

ZES represents a diagnostic challenge and portends a poor prognosis if not diagnosed and managed promptly. Although ultimately successful, the hospital course could have been expedited if the quality of the physician-patient relationship was higher. Special attention must be placed on identifying and overcoming compliance challenges to improve adherence. Clinical suspicion for ZES should be raised if there is a history of chronic diarrhea, GERD, and peptic ulcer disease. In our case, the diagnosis hinged on three key findings: abnormal EGD, elevated gastrin level, and a diagnostic SRS/SPECT. On EGD, hypertrophic gastric folds are a consistent feature of ZES, along with severe GERD and necrotic ulceration of the proximal duodenum, which may extend into the distal duodenum and proximal jejunum.
